# Performance of Rayleigh-Based Distributed Optical Fiber Sensors Bonded to Reinforcing Bars in Bending

**DOI:** 10.3390/s18093125

**Published:** 2018-09-16

**Authors:** Mattia Francesco Bado, Joan Ramon Casas, António Barrias

**Affiliations:** 1 Institute of Building and Bridge Structures, Vilnius Gediminas Technical University (VGTU), Saulėtekio al. 11, 10221 Vilnius, Lithuania; 2 Department of Civil and Environmental Engineering, Technical University of Catalonia (UPC), c/Jordi Girona 1-3, 08034 Barcelona, Spain; joan.ramon.casas@upc.edu (J.R.C.); antonio.jose.de.sousa@upc.edu (A.B.)

**Keywords:** structural health monitoring, damage identification, distributed optical fiber sensors, Rayleigh backscattering, reinforced concrete members, steel reinforcement bars, strain-reading anomalies, spectral shift quality

## Abstract

Distributed Optical Fiber Sensors (DOFSs), thanks to their multiple sensing points, are ideal tools for the detection of deformations and cracking in reinforced concrete (RC) structures, crucial as a means to ensure the safety of infrastructures. Yet, beyond a certain point of most DOFS-monitored experimental tests, researchers have come across unrealistic readings of strain which prevent the extraction of further reliable data. The present paper outlines the results obtained through an experimental test aimed at inducing such anomalies to isolate and identify the physical cause of their origin. The understanding of such a phenomenon would enable DOFS to become a truly performant strain sensing technique. The test consists of gradually bending seven steel reinforcement bars with a bonded DOFS under different conditions such as different load types, bonding adhesives, bar sections and more. The results show the bonding adhesives having an influence on the DOFS performance but not on the rise of anomalies while the reasons triggering the latter are narrowed down from six to two, reaching a strain threshold and a change in structure’s deformative behavior. Further planned research will allow identification of the cause behind the rise of strain-reading anomalies.

## 1. Introduction

### 1.1. Distributed Optical Fiber Sensors (DOFS) as a Tool for Structural Health Monitoring (SHM)

Structural Health Monitoring (SHM) techniques are finalized to the detection of deformation and cracking in concrete structures to ensure the safety of the infrastructure [1] by identifying early signs of excessive damage and giving feedback on the structure’s ability to continue serving its intended purpose. Henceforth, it crucially helps preventing the adverse social, economic, ecological, and aesthetic impacts that may occur in the case of structural deficiency. In the modern age, the most commonly used tools for capturing information on the structure’s health and efficiency are electrical strain sensors, accelerometers, inclinometers, GPS-based sensors, acoustic emission, wave propagation, etc. Yet, these methods have serious deficiencies among which the most significant are their sensibility-insufficient sensitivity to damage of applied methods, or to environmental disturbances [2]. More sophisticated optical fiber sensing techniques such as Fiber Bragg Grating (FBG) sensors [3,4,5] also have evident shortcomings among which are limited strain location range monitoring. These shortcomings prevent the possibility of precisely pointing out the location where the damage first occurs and prevents the linking of local damage mechanisms to the global condition of the structure [6]. This limitation is surpassed by Distributed Optic Fiber Sensors (DOFS). The fundamental principle of DOFS is the ability to measure strain and/or temperature along the fiber’s length by means of light scattering whenever the photons of the emitted light interact with the physical medium where this occurs (the fiber itself) and with local material characteristic features such as density, temperature and strain [7,8]. Three different types of scattering processes may occur in a DOF sensor, Raman, Brillouin and Rayleigh scattering, but all hold particular optical features that make one more suitable than others relative to the research objectives. While the Raman scattering is characterized by high dependence on temperature it can also be used to extract physical and chemical information of a material [9] such as food quality [10] or explosive materials [11]. Brillouin backscattering, thanks to its extended measurement range capability (up to several kilometers) is the most studied and used DOFS system in civil [12] and geotechnical [13,14] engineering. The third scattering phenomenon is Rayleigh scattering. Despite its 70 m sensing range limit, it can provide a high spatial resolution (up to 1 mm), which is ideal for strain and damage monitoring in concrete structures. The latter is often used in experimental laboratory investigations having the DOFS bonded either to its surface, to monitor its strains and damages [7,15], and/or on the reinforcement bars that will later be embedded in the concrete [7,16,17,18].

The distributed nature of such fibers enables the mapping of temperature, strain and vibration distributions in two or even three dimensions and their identification at any point along a fiber, henceforth allowing the painting of a clear picture of the global behavior of a structure rather than reporting the tensile state of a limited number of points [19]. This, in addition to its high accuracy, long-term stability, durability, and insensitivity to electromagnetic influences, corrosion, and humidity represents the great potential of the DOFS technique. Despite such, at the present moment the technique is still undergoing studying and research and therefore has not been standardized yet nor are there clear guidelines on how to ensure every deployment’s success.

### 1.2. Strains Reading Anomalies (SRAs)

**Figure 1:**
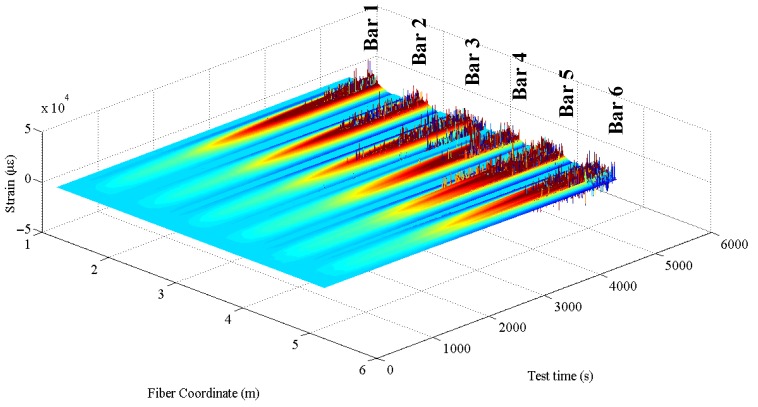
Three-dimensional representation of the strains measured by the DOFS over time along the fiber.

Two of the main points holding back the DOFS technique from becoming a truly universal and trustworthy strain-reading method are the frequent appearances of anomalies in the DOFS measurements and the absence of a reliable DOFS/structural support bonding technique. Multiple publications based on DOFS-monitored experimental campaigns report coming across strain-reading anomalies (SRA) in the form of localized and excessively large strain peaks [16,17,18,20] which have seemingly little to no physical meaning or explanation. For instance, the DOFS may report in a single point of the fiber large tensile strain peaks followed by equally large compressive strain peaks in the span of a few seconds. These anomalies do not seem to be restricted to a particular model of Optical Backscatter Reflectometer (OBR) as they seem to rise up in both ODiSI-A model [15,16] and OBR 4600 [17,18,20], both manufactured by LUNA Technologies (Roanoke, VA, USA). This situation occurs also in the experimental test described in this paper as shown in Figure 1. Such readings can be considered abnormal for the following reasons:

- their sheer size
- the large difference with the previous readings despite the load difference between the two being almost negligible
- the occurrence of large compressive peaks in a homogenously tense area of the structure

This acute proximity among tensile and compressive strain peaks can also be present geometrically speaking. Indeed, such opposite strain peaks may be reported in neighboring sections of the monitored structure even a few millimeters distant. This is abnormal considering that usually both sections are entirely and homogenously in tension or compression and further illogical considering their co-existence at a very short distance from one another. The phenomenon here described has little to no physical meaning suggesting that it might simply be an erroneous DOFS strain-reading. This assumption is confirmed when comparing the monitored structure’s behavior under loading with theoretical models and simultaneous strain gauges monitoring. In the present case, none of the above methods concurs with the DOFS’s reported strain peaks suggesting that these are in fact SRAs. These anomalies should obviously be avoided as they prevent the possibility of getting reliable measurements in the single location of the anomaly (time-wise and geometrical-wise anomalies) and extensively increase the level of unreliability of the surrounding measurements.

Despite the frequent occurrence of such phenomenon in DOFS-monitored civil engineering-oriented experimental campaigns, it has been the topic of little research and hardly any data is available on its possible origin. Among it, the authors of [21] offer an essential take on the subject. Indeed, according to the latter, the DOFS reading anomalies are mainly determined by local failure of the algorithm that tracks the beating between the local oscillator point of the range under analysis in the OBR software and the light from the fiber during the wavelength sweep. This may further be due to the un-altering of factors such as the sensor gauge length and spacing. Yet, this is the case for homogeneous materials subjected to low strain values; however, when dealing with substrates of more heterogeneous composition (such as concrete) or/and with strong discontinuities (cracked RC members), it may be different as these physical elements and mechanisms may affect the fiber’s readings.

The present paper discusses the results of an experimental campaign exploring strictly the so-called physical causes of these anomalies in the case of DOFS subjected to high levels of strains or bridging local discontinuities of the substrate material, with the objective of removing the consequent physical-induced anomalies from the extracted data. Oppositely, issues connected to the OBR’s intrinsic algorithms and quality of the collected Rayleigh shift later used for the correlation, are not contemplated in the present publication. Lastly, the paper aims at drawing some conclusions regarding the efficient use of DOFS in the case of civil engineering structures monitored by means of commercially available OBR systems as the one used in the tests herein reported.

## 2. Experimental Investigation of the Strain-Reading Anomalies

### 2.1. Test Motivation and Setup

**Figure 2:**
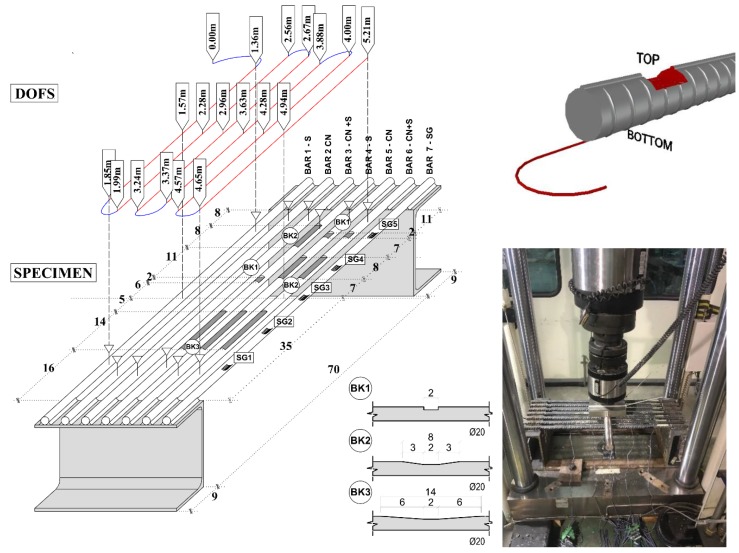
Test setup and three-dimensional specimen detailing with vertical DOFS representation.

The authors have conceived an experimental test aimed at pin-pointing the motive behind the SRAs. The test intends on inducing these in a controlled environment under different conditions to isolate and identify the cause of their origin. As presented in Figure 2 the experiment, carried out in UPC-BarcelonaTech’s laboratory, sees the loading of seven parallel S500 steel reinforcement Ø20 bars with their extremities welded to two C shaped steel beams (assuming a fixed beam behavior).

Six of the rebars are instrumented with a single 5 m long DOFS (Bars 1–6) and one is instrumented with strain gauges for comparison purposes (Bar 7). Please note that, while the current DOFS is commercially considered as a 5 m long fiber, its sensing length is slightly longer (5.21 m) as visible in all the extrapolated data. To instrument multiple bars with the same DOFS, the latter has some segments bonded to the lower face of each bar (providing strain measurements and drawn in red in Figure 2) and others that function merely as bridges between the first ones (these do not yield any useful strain measurements, drawn in blue). Figure 2 indicates in the three-dimensional model of the specimen what are the segments of each bar instrumented with the DOFS as explained in the following; two white triangles on every bar indicate the first and last point that the fiber is bonded to the rebars signifying that all those in between are being monitored. Table 1 indicates which points along the 5 m long fiber correspond to these triangles, in other words, providing their DOFS coordinate. The table and Figure 2 also include the DOFS coordinates of those points where the INSTRON actuator is in contact with the rebars, loading them.

For example, the strain readings extrapolated by the DOFS coordinate 1.42 m refers a particular section in Bar 1. Oppositely, the readings from the DOFS coordinate 1.89 m can be disregarded as they report the strains from a section of the DOFS that is not glued to any rebar.

The current research uses the Optical Distributed Sensor Interrogator (ODiSI-A) which analyzes the Rayleigh backscatter from a standard single-mode DOFS set to measure strains and temperature. The test surveys the DOFS performance for different bonding conditions, rebar sections and loading speed. Indeed, two kinds of adhesives were used to bond the DOFS to the bars. Cyanoacrylate for Bar 2 and Bar 5; silicone for Bar 3 and Bar 6; cyanoacrylate with an extra silicone layer on top for Bar 1 and Bar 4. Furthermore, Bars 4, 5, 6 were incised along their upper face to locally reduce their sectional area. The dimension of such incisions can be read in Figure 2. Some incisions are very sharp meanwhile others are smoother. The bars are loaded simultaneously in their mid-span with different speeds and with two impact loads. The latter stands for loads applied on the specimen in the fraction of a second and are designed to be included in the loading plan between steps of monotonic loading.

As mentioned earlier, the test studies multiple scenarios that could be the physical cause of the strain-reading failures SRAs. These scenarios will henceforth be called Disruption Mechanisms. Below are listed seven of these, each followed by the experiment’s attempt at simulating them.

(DM-SB1) The bending of the bar (the fiber’s support) beyond a certain point may be too high for the tensile and shear strength of the fiber’s constituting material therefore causing SRAs in the produced readings. The existence of a strain value beyond which anomalies are consistently more susceptible to be triggered is herein defined as Anomalistic strain threshold (AST). The veracity/influence of such disruption mechanism will be examined by applying an increasingly larger bending load on the rebars while constantly monitoring the strain profiles provided by the DOFS on the lookout for the appearance of SRAs.

(DM-SB2) When the first crack appears in a bending RC member, the stress that the tensile rebar suffers in the cracked section increases exponentially (the strains could be even 20 times larger than its previous value). Indeed, the steel suddenly passes from simply contributing to resisting the local tensile stress concurrently with the concrete (when the section is whole) to bearing it alone (having the concrete cracked). The resulting abrupt peaking of stresses in the steel rebar will automatically be transferred to the bonded DOFS which could potentially lead to the disruption of its reading capacity. To simulate such, two impact loads are applied on the bars during the test. If the fiber can deliver reliable readings after having suffered these impacts, then it can be assumed that DM-SB2 does not affect the DOFS’s performance. It should be noted that, if the increase in stresses in the rebar when concrete cracks were to be entirely simulated in the present test, it would lead to a very large bending of the rebars and of the DOFS. In a RC member, being the rebars restrained by the surrounding concrete, such hefty bending would not occur making the latter excessive and unrealistic. Furthermore, if SRAs would spring up after such impact, it would be unclear whether they were caused by the sudden rise of strains in the fiber or by the sudden increase in curvature making the test inconclusive. The impact loads are, therefore, limited to 0.1 kN and 1.0 kN causing in each bar an increase of respectively 1.6 MPa and 16.0 MPa.

(DM-SB3) In the aftermath of the cracking of a bending RC member, a redistribution of stresses and strains occurs inside it. According to the stress-transfer laws for cracking RC members [22], the steel rebar’s new strain profile boasts a peaking of stresses in correspondence of the cracked sections while it stays unaltered in the sections most distant from the cracks. This is due to stresses being transferred from concrete to steel the closer the case-study section is to the cracked section. How quickly such stresses are transferred is a function of the transmission length *l_t_*. An excessively quick transfer (corresponding to a short *l_t_* on either side of the cracked section) may cause errors in the DOFS readings. To simulate different transmission lengths, the experimented rebars present incisions with steep or inclined/smooth edges. The reduced area of the rebars leads to increased carried stresses in the incised sections, similarly to what happens in RC beam sections that have cracked compared to those that have not. A steep edge of the incision (Block 1) can be compared to a short transmission length *l_t_* while a sloping one (Block 2, 3) can be compared to a long *l_t_*. The strains in these three rebars are closely monitored to verify whether a drastic section gradient (Block 1) causes SRAs first compared to softer incision edges.

(DM-AD1) According to some publications on DOFS-based research [14,18], SRAs seem to spring up soon after the appearance of the first concrete damage sign suggesting that there might be a connection between the two. Taking in consideration a RC member with DOFS-instrumented rebars, if the adhesive positioned on top of the fiber is not protective enough (not thick enough or composed of excessively deformable material), then the cracking concrete friction against the fiber may cause alterations in it leading to SRAs. While DM-AD1 cannot be checked with the present test, being it deprived of concrete, it is nonetheless possible to check the difference in the strain-reading capacity of the DOFS when it is covered by a single layer of adhesive or double (having respectively a bonding and protective function). This is the reason cyanoacrylate and an extra silicone layer on top are deployed in Bars 1 and 4.

(DM-AD2) To a certain extent, being completely embedded in adhesive, the DOFS reports as much latter’s strains as the supporting structure’s. Under the hypothesis of perfect bond between adhesive and supporting structure the strains are identical, but this may not always be true. When adhesives harden, they deliver their top stiffness performance ensuring the strongest bond with the structural element, but they may also acquire deformative features (fragility, segmented behavior, etc.) that could lead, when under stress, to their delamination and cracking altering the performance of the embedded fiber. During the loading phase the adhesives are monitored to spot eventual deterioration.

(DM-AD3) An incorrect DOFS confinement design may lead to altered strain readings. The use of an adhesive with poor confining strength properties or the application of an insufficiently thick layer may cause the fiber to delaminate from its support, therefore losing the hypothesis of perfect bond with its support. As before, the adhesives are monitored during the test to spot any delamination.

(DM-AD4) It is possible that the viscosity of the adhesive (significant when using silicone) influences the fiber’s performance ensuring a minimum amount of slip between the fiber and the adhesive. This allows a minor stress redistribution among the neighboring fiber sections that could be the key in avoiding premature SRAs despite being detrimental to the precision of strains measurement. A comparison between the readings provided by the portion of DOFS bonded with cyanoacrylate is compared to that coming from the portion bonded with silicone.

### 2.2. Test Procedure and Outputs

The results reported by the DOFS are studied keeping in consideration the following aspects:

- the specimen’s rebars were not exactly on the same plane due to slight deformations occurring during the welding process. As a result, the applied load, herein defined as global load, is not evenly distributed among the rebars. Automatically some of them suffer more strains than others.
- As a consequence of the point above, the bars suffering larger strains are the ones reaching first the yielding stress. In that instance, only some bars have yielded while the others have not. Later in the test, with the increase of the global load, every bar eventually reaches its yielding point.
- Bar 1 behaves differently than the other six. Indeed, notwithstanding the other rebars do not precisely behave as end-fixed beams, as seen later, their behavior gets rather close. On the other hand, Bar 1 seems to have hardly any stiffness at its extremities and, because of such, does not seem to participate to the structure’s rigidity.

**Figure 3:**
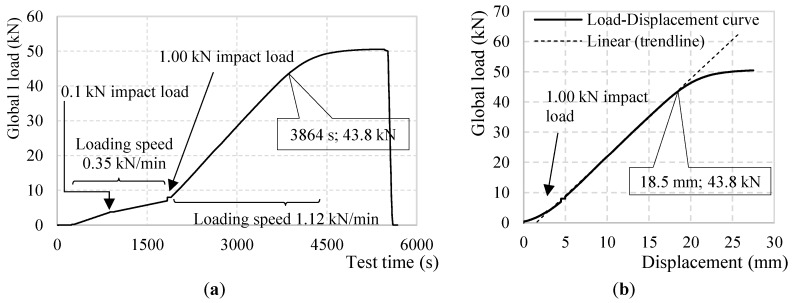
(**a**) Test load curve over time and (**b**) over the load actuator’s-measured displacements.

As mentioned above, during the test the specimen is subjected to two loading speeds and two impact loads. Initially the specimen is loaded at a speed of 0.35 kN/min. At 880 s an impact load of 0.1 kN is applied followed by a 60 s hold. At its conclusion, the specimen is again loaded at 0.35 kN/min until, at 1835 s, a second impact load (1.0 kN) is applied. As before, a 60 s hold follows, at the end of which the specimen is loaded at a quicker speed of 1.12 kN/min until the specimen’s plasticity is reached. The load-displacement curve, as obtained by the INSTRON hydraulic actuator, is presented in Figure 3.

**Figure 4:**
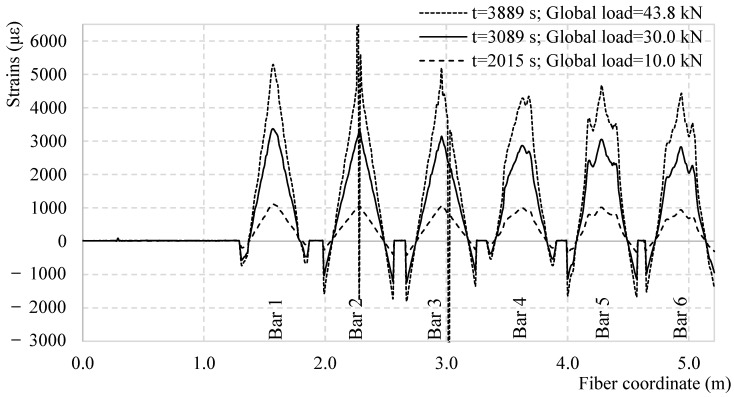
DOFS strain profiles at specific time and global load instances along the test.

With this in mind, the DOFS global strain diagram is represented in Figure 4 for three increasing load levels. The graph represents the strain distribution along the whole 5.21 m long fiber at specific instances and specific global load values. Each of the graphs’ six visible tensile peaks represents the strains in each of the six bars that are DOFS-instrumented. As example, in the strain profile extracted at 2989 s, corresponding to a global load of 28.18 kN, it can be stated that the mid-span of Bar 1 is characterized by a strain state of 3165 με. On both sides of each tensile peak are compressive measurements that do not represent the entirety of the compressive portion of the bars’ strain state as the DOFS is not glued all the way to the supports. Finally, the strain-less horizontal segments between the compressive segments are the above-mentioned sectors of the DOFS that act as bridges between the segments bonded to the bars. The uneven distribution of the global load among the seven bars is clear when comparing the maximum strain suffered by each. Finally, if to consider the strain profile at 3889 s, at an applied global load of 43.82 kN, some SRAs are visible (mid-span of Bar 2 and Bar 3) making the readings impossible in those areas.

**Figure 5:**
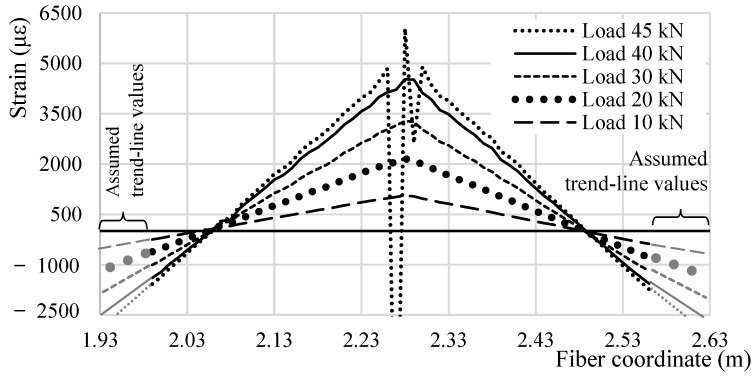
DOFS strain profiles at specific time and global load instances along the test in a specific segment of the DOFS bonded to Bar 2.

**Figure 6:**
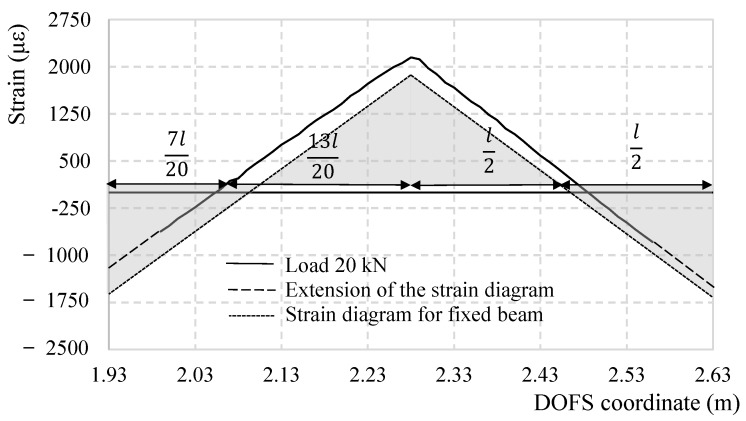
Comparison between the strain profile corresponding to a global load of 20 kN of a theoretical end-fixed specimen and the DOFS measured one in the segment corresponding to Bar 2.

**Figure 7:**
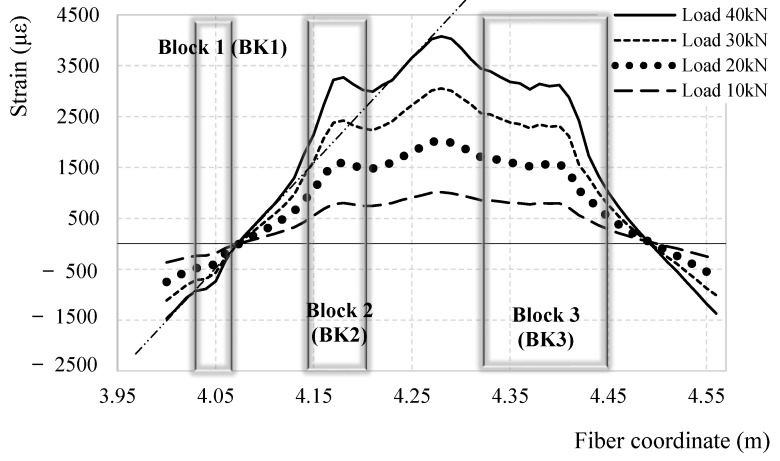
DOFS strain profiles at specific time and global load instances along the test in a specific segment of the DOFS bonded to Bar 5.

If to focus on the strain profile of an individual rebar at different global load levels the result would resemble Figure 5 for Bar 2. As noticeable its profile seems to grow in a very reasonable and reliable fashion up until the appearance of the first SRA (in this case between 40 and 45 kN), after which some values are un-readable. On both extremities, the strain profile in compression is plotted until the support following the strain’s trend-line and assuming the continuity of linearity. By plotting these, it is evident how, as represented in Figure 6 (for a global load of 20.0 kN), the behavior of the rebar does not perfectly reflect the behavior of a fixed beam (represented in grey). Truly, the supports are not perfectly rigid in front of rotation, leading therefore to higher strains in the mid-span and lower at the extremities if compared to a perfectly clamped beam. This leads to the mid-span section to reach the yielding stress earlier than the supports. Figure 7 plots the strain profile of a singular rebar (Bar 5) except in this case some incisions are present along its length. The strain profile’s loss of linearity in the specific segments is congruous with the rebars’ sections that have been incised making them easily recognizable on the measured DOFS strain profile. Before and after the incision’s profile variations, the linearity is re-acquired. This figure also demonstrates the DOFS ability to read strains all along Bar 5 free of SRAs (until later in the test when many sections acquire SRAs) despite the presence of incisions with and without steep edges. This discards (DM-SB2) from the possible disruption mechanisms.

**Figure 8:**
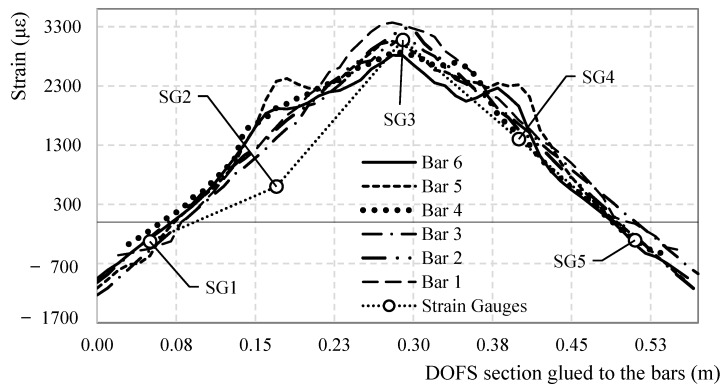
Comparison between the DOFS-measured strain profile segments bonded to each rebar for a global load value of 30.0 kN.

Comparing the strain profile of all the seven rebars at a global load of 30.0 kN (3089 s), as in Figure 8, the similarity among them is evident (except for the local increase in strain in the location of the incisions) along with the correspondence between the strain gauge’s and the DOFS’s measurements. Strain gauge 2 seems to be the only one not reporting correct values, probably due to a slight damage occurred during the positioning process. This embodies one more advantage of the DOFS. Indeed, differently with the strain gauges, the latter does not require fixing of every single sensing point, making it so that the correct positioning of the fiber is enough to ensure the reading capacity and reliability of each point. The comparisons among each bar’s DOFS strain profile along with their coherence with the strain gauges’ measurements is a testament to the performance and the reliability of the DOFS strain sensing technique.

## 3. Discussion of the Results Regarding SRAs

### 3.1. Analytical Definition of an SRA

According to the DOFS provider’s user guide [23] (LUNA Technologies), the reliability of all strain readings can be assessed through their associated Spectral Shift Quality (SSQ) value. The SSQ is a qualitative measure of the correlation strength between the conducted measurement (at any point and time) and the original baseline reflected spectra as in Equation 1 below.

(1) $$\mathrm{SSQ}=\frac{\max(U_{j}\left(v\right)*U_{j}(v-\Delta v_{j))}}{\sum U_{j}{\left(v\right)}^{2}},$$

with:

- *U_j_*(*v*) the baseline spectrum for a given segment of data
- *U_j_*(*v* − ∆*v_j_*) the measurement spectrum under a strain or temperature change
* the symbol is used to represent the cross-correlation operator

**Figure 9:**
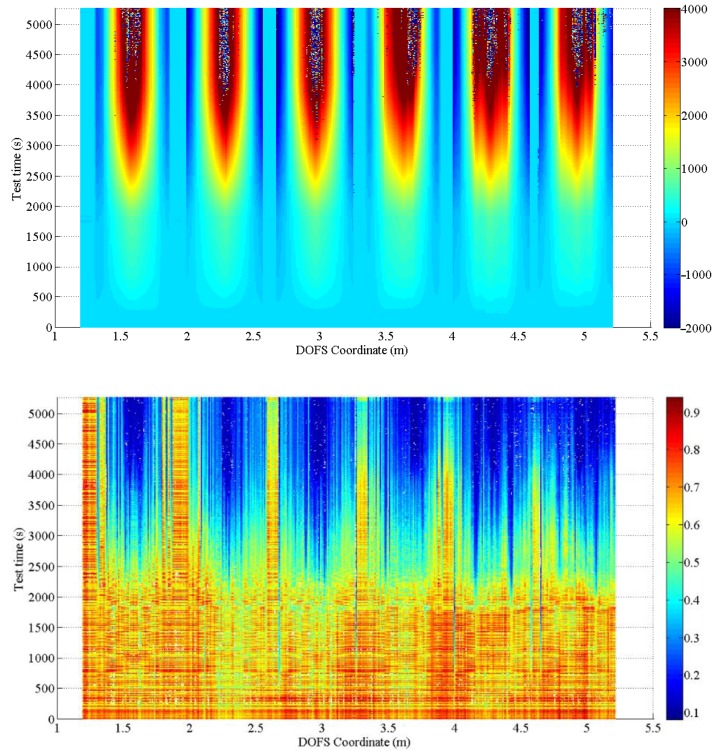
Two-dimensional representation of DOFS-measured strain along time over the whole span of the fiber bonded to the rebars (**above**) and their relative SSQ values (**below**).

Theoretically, the Spectral Shift Quality should be a value between 0 and 1, where 1 is a perfect correlation and zero is uncorrelated. Practically talking though, the calculated value can exceed 1 due to small variations in the laser power. The data sets will be accurate if the Spectral Shift Quality is above 0.15. Instead, if the SSQ is less, it is likely that the strain or temperature change has exceeded the measurable range. Please note that, as strain or temperature variation increases, the Spectral Shift Quality will decrease. The SSQ automatically qualifies as a tool to gain insight on the accuracy of every strain-reading and identification of SRAs [18]. Figure 9 provides a graphical depiction of the values of SSQ corresponding to each strain measurement performed by the DOFS in the test.

**Figure 10:**
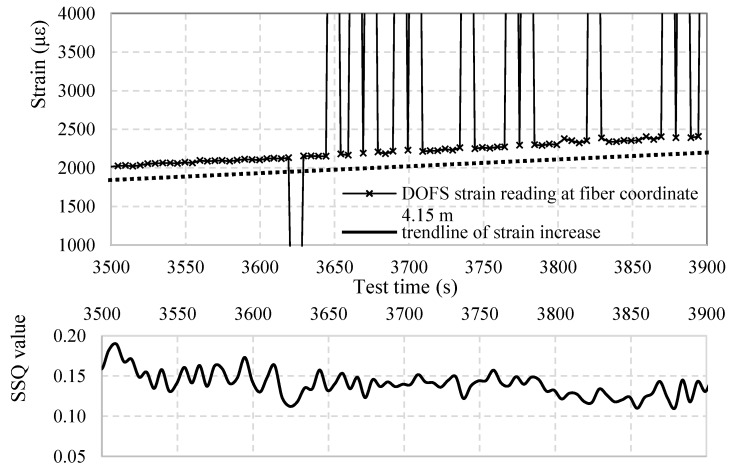
DOFS-measured strain of the fiber coordinate 4.15 m along time (**above**) and the corresponding SSQ value of each measurement (**below**).

Yet, according to the latter, the more the fiber is under stress the less the SSQ seems adapt to the identification of anomalies. Figure 10 provides a good example of such. In its upper part is shows the strain evolution in time of the DOFS coordinate 4.15 m with below its corresponding SSQ values. Every X marker along the plotted line represents a single measurement developed in time while the peaks are the graphical expressions of SRAs. Below are the corresponding SSQ values of those same DOFS readings. Evidently, between 3500 and 3620 s no strain measurements should be considered anomalistic as they all clearly plot the evolution of strains in the DOFS coordinate. Yet, the associated SSQ value of these readings drops multiple times below the threshold of 0.15 suggesting that at least half of such measurements are indeed anomalistic. This is obviously false. The SSQ value seems to start faulting in its SRAs identification function the closer the measurements get to the first anomaly. The same can be said for the segments in between SRAs. Indeed, in between 3790 s and 3850 s the corresponding SSQ value of the strain measurements is never above 0.15 despite its profile indicates the presence of very realistic and reliable measurements. Furthermore, the point of this interval with the highest SSQ value actually corresponds to the SRA itself (it should have the lowest SSQ value). In conclusion, while the SSQ can prove to be a good preliminary tool for identifying anomalies, it is does not meet the accuracy requirement for identifying the SRAs present in the current test. To compensate for such, the authors suggest a simpler and more efficient way of identifying SRAs. Given that, by definition of SRA, they correspond to geometrical discontinuities of the Strains/Time or Strains/DOFS coordinate diagrams it is sufficient to set a specific value of strain increment that, when compared to the difference of strains values between two consecutive measurements, if surpassed it indicates the presence of an anomaly. In the present research such value was set to 200 με.

**Figure 11:**
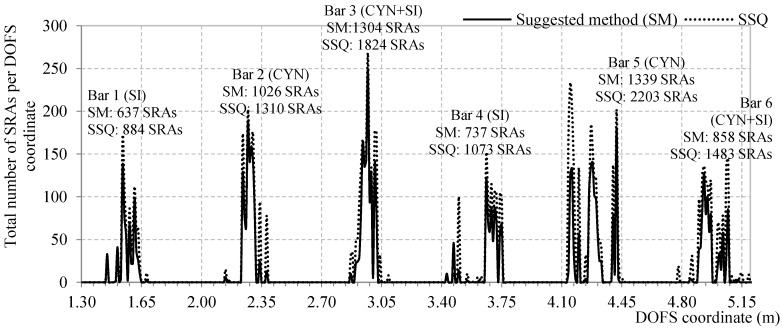
Sum of total number of SRAs for every DOFS coordinate for the duration of the test.

In continuation, Figure 11 plots the total calculated number of SRAs per each DOFS coordinate calculated both by means of the SSQ and with the above-mentioned methodology (SM). The method that uses SSQ over-estimates by 30% the number of SRAs henceforth discarding 23% of strain readings that would be completely readable and able to provide consistent insight in the strains of the support structure.

### 3.2. SRAs Mechanics

**Figure 12:**
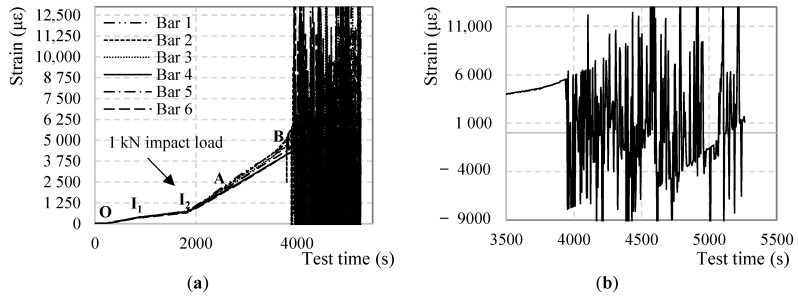
(**a**) DOFS-measured strains of the mid-span of every bar along time (**b**) zoom on Bar 3’s anomalies.

Figure 12 represents the measured strain in six points of the fiber, corresponding to the mid-spans of the six bars, from the first moment the bars were loaded (point O) until the end of the test including the instances at which the impact loads were applied (I_1_ and I_2_). After around 3800 s (Point B) the profiles are completely dominated by SRAs making it impossible to extract any reliable strain values.

**Figure 13:**
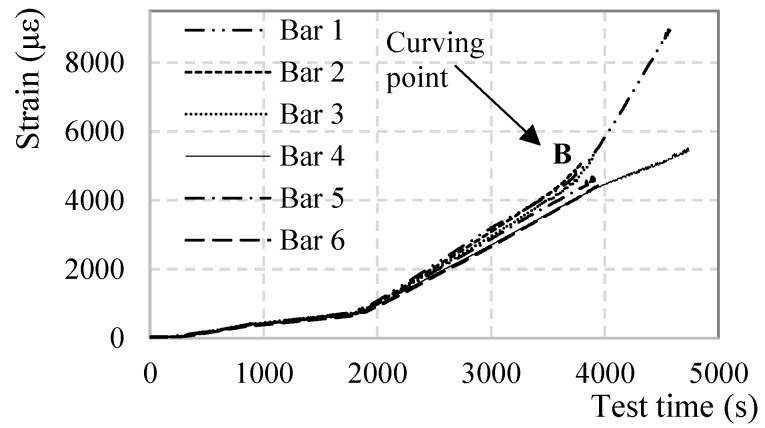
DOFS-measured strains of the mid-span of every bar along time without depiction of SRAs.

Several considerations can be developed regarding Figure 12:

- The strain profiles until point B and measured strains lower than 4000 με are clear and SRA-free.
- All segments of the DOFS have successfully recorded both impact loads demonstrating their ability to correctly withstand and record a sudden stress growth of 1.6 MPa and 16.0 MPa, as designed by (DM-SB2).
- The strain profiles do not report a change of linearity beyond the steel yielding strain, occurring in the mid-span, as it would be expected. ε_s,y_ for an S500 steel corresponds to 2500 με (reached at around 2800 s according to the above Figure 12, point A). This agrees with Figure 3 which also does not seem to report any curvature at around 2800 s.
- The strain profiles report correctly and precisely the variation of loading speed, both before and after the yielding of the steel rebars.
- The strain profiles correspond well with the applied load despite them being slightly offset compared to one another. The reason behind it is the difference among the heights of the bars when the specimen is unloaded and the consequent uneven distribution of the global load among them.
- Figure 13, clears out the SRAs from Figure 12. The different bars seem to start curving at point B which, once again, agrees with Figure 3.

As pointed out above, despite all rebars having yielded in the mid-span, the DOFS strain profiles of Figure 13 do not exhibit any evident curving until point B. Here is when the fixed end of the rebars also yield turning into plastic hinges herein the definition of point B as Plasticity point. The latter does not happen simultaneously for all the bars due to the uneven load distribution among them. It is, instead, scattered in time. Only once the last of the six bars acquires plastic hinges (Bar 4 at 3864 s), the load/displacement and load/strain diagrams begin to clearly deviate from linearity. It is noticeable in Figure 4 how the fixed edges of Bar 1 are strangely less stressed than all the others and hence far from yielding during all the duration of the test. This inconsistency suggests the failure of Bar 1 in acting as a proper end-fixed beam therefore not contributing to the rigidity of the structure. So much so that the load-displacement curve deviates from its linear trend when the extremities of Bars 2–6 yield, completely unaffected by the fact that Bar 1 is, oppositely, far from it.

**Figure 14:**
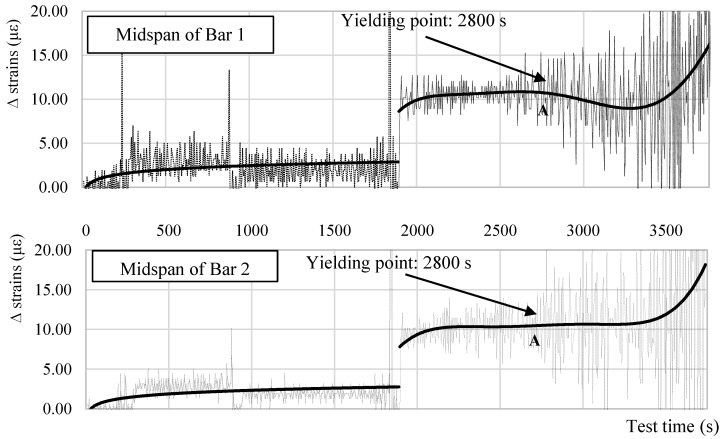
Strain increment ∆ for the mid-span of Bar 1 and Bar 2 along time.

On another note, Figure 13 is unclear about the instance/strain at which the steel yielding occurred. To visualize it, Figure 14 plots the strain increment ∆ strain at each DOFS measurement in the mid-spans of Bar 1 and Bar 2 as examples of the behavior in all bars. The graphs plot only the ∆ strain from 0 s to 3750 s as, beyond such, the anomalistic values would make it impossible to calculate a realistic trend-line. Up until the yielding point (point A) the trend-line of the strain increments are mostly linear (excluding the parts straight after the beginning of the test and straight after the impact load when the DOFS must “settle”). Beyond this point, the linearity is lost therefore confirming the yielding of the bars in the mid-span occurring around 2500 με.

Back to Figure 12, the SRAs seem to be triggered after point B which sees the change in deformative behavior of the specimen and therefore its loss in linearity of the load-displacement diagram. On the other hand, the anomalies seem to be initiated in the proximity of a value (around 4000 με) that could represent the anomalistic strain value AST. The points in defense of the latter option will be discussed later.

**Figure 15:**
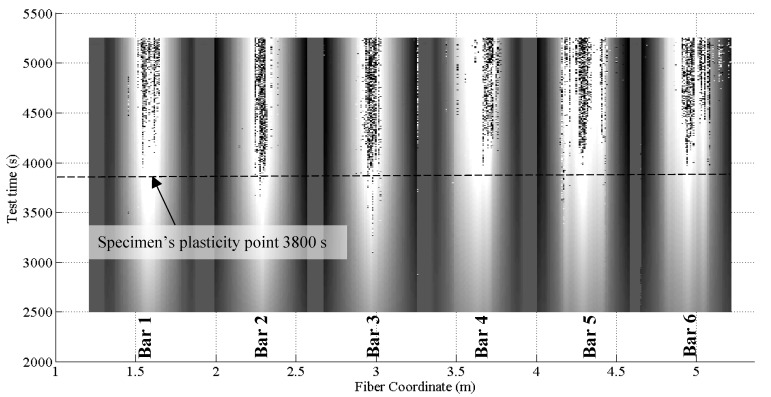
Two-dimensional representation of DOFS-measured strain along time over the whole span of the fiber bonded to the rebars and of the SRAs.

**Figure 16:**
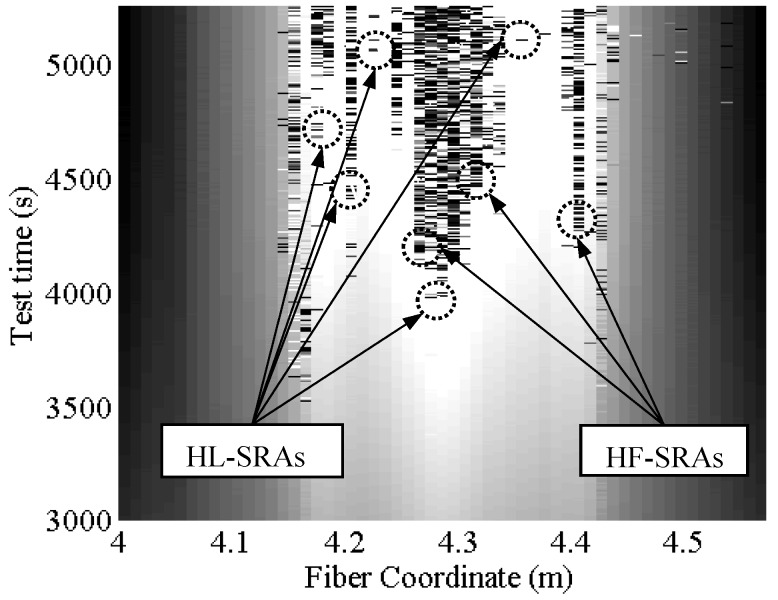
Two-dimensional representation of DOFS-measured strain along time over the span of the fiber bonded to the rebar 5 and example of distinction between harmless and harmful SRAs.

**Figure 17:**
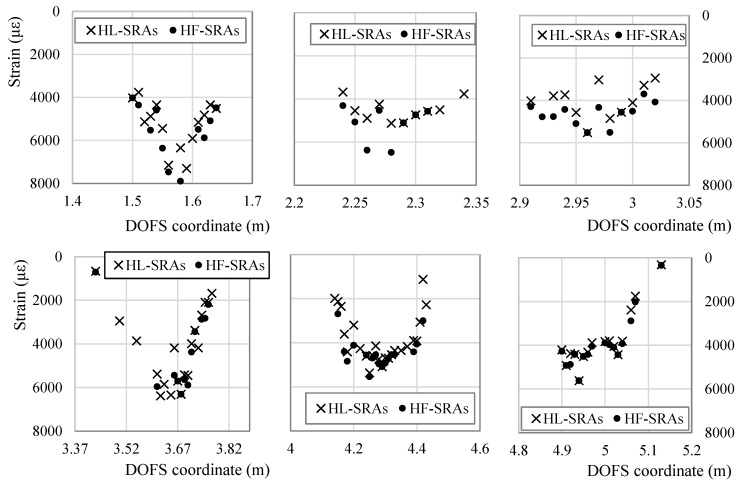
Plotting of DOFS-measured strain values at which the first HL-SRA and HF-SRA are recorded in each DOFS coordinate with anomalies.

SRAs do not rise strictly in the mid-span of the rebars and do not necessarily rise there first either. Figure 1 and Figure 15 are a 3-dimensional and 2-dimensional graph representing the DOFS-measured strains in the six bars versus the test time. In Figure 1 the SRAs can be identified as perpendicular peaks coming out of the graphed surface while in Figure 15 they can be identified as black bars. Both graphs allow not only the location of the SRAs in time but also the geometric correlation with the section of the rebars. Figure 16 zooms in Figure 15 to focus strictly on Bar 5 and reports the strain values at which the SRAs occur. A quick glance of it shows the existence of SRAs that are followed by additional anomalies for all the remaining duration of the test while others that, despite preventing the correct reading of the strains at a specific instance, still allow it later. Other SRAs instead are followed by additional anomalies for all the remaining duration of the test. It is worth distinguishing the two as one represents the end of any reliable strain measurements in a specific section Harmful Anomalies (HF-SRA) while the other Harmless Anomalies (HL-SRA) do not. Figure 17 plots, for anomalistic DOFS coordinates, the strain values at which the first HL-SRA and HF-SRA spring up therefore giving a graphical representation of the SRAs/strains correlation.

Some interesting observations can be developed based on such graphs:

- Except for a few DOFS coordinates, the SRAs start beyond the specimen’s plasticity point reached around 3800 s.
- SRAs are often concentrated in specific areas where all neighboring sections give evidence of anomalistic behavior. Such areas can be defined as Anomalistic areas.
- Averagely SRAs seem to spring up from the mid-span of the rebars first which, not coincidentally, are the most stressed points of the seven rebars. The anomalies later spread outwards towards the neighboring section (clear in Bars 2, 3, 6) forming an anomalistic area.
- In Bars 4, 5, 6, the SRAs also rise in the incised sections concurrently or soon after having appeared in the mid-span (particularly evident in Bar 5). This further confirms the hypothesis of SRAs springing up from highly stressed rebar sections.
- HF-SRAs are usually proceeded by HL-SRAs.
- In some rebars HL-SRAs seem to start slightly before the specimen’s plasticity point while all HF-SRAs after that. In particular, Bar 3 starts having HL-SRAs as soon as 3000 s at DOFS coordinate 2.97 m.
- In some cases, a DOFS coordinate can be characterized strictly by HL-SRAs, always guaranteeing intervals of strain readability such as DOFS coordinate 4.16 m in bonded to Bar 4.

As evident from Figure 16 and Figure 17, on the edges of the anomalistic areas, the SRAs spring up at lower and lower strains the more the DOFS coordinates are distant from the mid-span of the rebar. This could debunk the possibility of the existence of an AST. On the other hand, it can still be plausible considering the presence of the following phenomena. Assuming the existence of a contagious influence that an anomalistic fiber section could pass on to neighboring ones, then it would be sufficient that any DOFS coordinate suffered of the first SRA to start a chain reaction that would lead to the creation of anomalistic areas. This would prevent the sections included in such area to reach the AST being their SRAs triggered prematurely.

Another point in favor of the existence of an AST is suggested by the location of most HF-SRAs in Figure 17. Indeed, 83% of HF-SRAs are triggered at a strain level beyond 4000 με while the other 17% are HF-SRAs that have sprung up on the edges of anomalistic areas. As explained above, the smaller portion of HF-SRAs are probably triggered prematurely by the influence exercised by the anomalistic behavior of neighboring sections which already have SRAs. Table 2 attempts to extrapolate the value of AST differently. It takes in consideration all the SRAs along the whole DOFS and calculates their total number beyond a specific value of strain. In the presence of an AST it should be possible to see in Table 2 an increase of total anomalies beyond a specific strain value. As a matter of facts, around 4500 με the curve suddenly becomes steeper suggesting that the latter is the AST. Between the two ways of calculating the AST, the first is not only more conservative but also tackles the issue of HF-SRAs which represent the biggest threat to the monitoring of an experimental campaign.

In conclusion, the SRAs seem to proliferate on all six DOFS-instrumented bars in the most stressed sections (mid-span or/and incisions). This occurred late in the test both in full and incised sections and despite the early application of two impact loads. Consequently, DM-SB2 (for small impact loads) and DM-SB3 can be discarded as potential triggers of the SRAs. Harmless anomalies HL-SRAs rise earlier than Harmful ones HF-SRAs but, being the proliferation of the latter more troublesome in experimental tests, they are the focus for the determination of an AST. The value of the latter is 4000 με (DM-SB1) which, unfortunately, coincided time-wise (3800 s) in the test with the specimen’s change in deformative behavior. The coincidence of these two phenomena prevents the pin-pointing of which of these two factors is the actual SRA trigger. Further experimental investigation is therefore required.

### 3.3. Adhesives Performance

**Figure 18:**
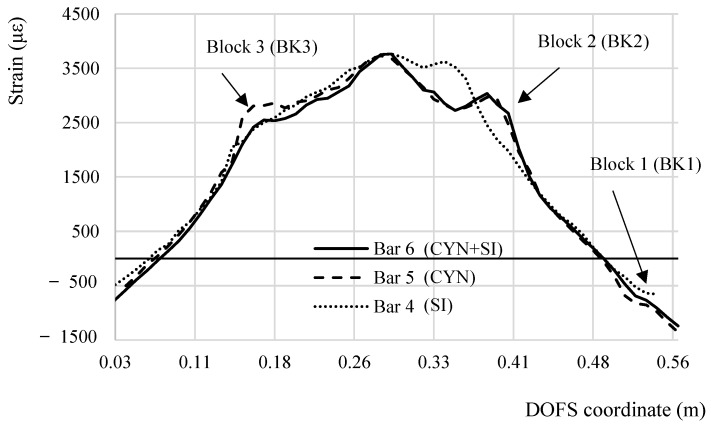
Comparison between the DOFS-measured strain profile segments bonded to Bars 4, 5, 6 for a global load value of 30.0 kN.

In the present section the performance of the different adhesives used in the test is analyzed to extract conclusions over which is most suitable for DOFS-monitored rebars embedded in RC members. To bond the DOFS to the rebar silicone (SI), cyanoacrylate (CYN) and a combination of the two (CYN+SI) have been used respectively in Bars 1, 4 and Bars 2, 5 and Bars 3, 6. Figure 8 compares the strain profiles obtained by all of them at a specific global load with an evident good agreement. The same cannot be said in the incised sections for Bars 4, 5, 6. Figure 18 compares the DOFS strain profile of these three bars after having equalized the maximum strain (3700 με). Bar 5 and Bar 6 share the incision layout (as evident in the specimen dimensions in Figure 2) while Bar 4 shares the layout of the other two only on half of its length (left half of Figure 18). The decision behind this different layout was taken to check whether a steep-edged incision in proximity of the point that supposedly suffers the harshest stresses (the mid-span), would be the birth-place of the DOFS’s first SRAs.

The following observations can be listed following an analysis of Figure 18.

- All three strain graphs are in very good agreement in their linear sections and in the mid-span.
- Bar 5 and Bar 6 are in excellent agreement on the strain distribution around Block 2, slightly less but still reasonably around Block 1 and differently in Block 3.
- Around Block 3 similar strain values are reported by Bar 4 and Bar 6 (despite their curve profile is not matching). Both include silicone while Bar 5, the only bar providing a different strain value, includes cyanoacrylate.
- Bar 5 and Bar 6, despite providing different strain values, have a similar curve profile especially around Block 3 where a “strain bump” is present. Differently, on the left side of the graph, Bar 4’s profile is almost linear.

The above listed points can all be explained taking in consideration the constitutional property of the silicone and its easily deformable and elastic nature even after curing. When the DOFS support (the rebars) deforms, the fiber does not deform in an equivalent manner because of a quantity of slip allowed by silicone. This slightly redistributes it among the neighboring sections reducing the stresses on the fiber in Block 3 and leading to the transformation from a strain peak to a lesser and wider “strain bump”. In the case of Bar 6, the two adhesives act as a composite material displaying some behavioral features of one and the other. Indeed, in Block 3 its strain profile shows a peak which, despite being of smaller quantity than Bar 5’s, is still evidence of the cyanoacrylate behavior. The reduced entity of Bar 6’s peak can be explained by a redistribution of stresses that the silicone intrinsically performs when receiving the stresses from its contact surface with cyanoacrylate. If there were not any slip between the supporting structure and the adhesive and between the adhesive and the DOFS, for a continuous fiber the strain in the latter would equal the strain in the supporting structure. For silicone, though, the slip is present, slightly altering the strain readings on the bar oppositely to the cyanoacrylate that, without slip, accurately reports its strains. From a strain fidelity point of view, the cyanoacrylate is the best adhesive. On the other hand, whenever using silicone for DOFS bonding, the correct position of the incisions on the rebar (which in real RC members would be the cracks) is correctly detected. On the other hand, Bar 5 and Bar 6’s behavior is perfectly equivalent in Block 2 and Block 1 suggesting that the redistribution of silicone is a function of the transmission length *l_t_* If the latter is short enough, hardly any redistribution occurs while for larger ones, such as Block 3, the redistribution can fully develop.

Figure 11 plotted the total amount of SRAs that occur in all DOFS coordinate for the duration of the test up until a global load of 49.8 kN (end test is at 50.5 kN) and indicated what was the total number of SRAs in each segment of the fiber bonded to each bar. The rebars having the least number of anomalies are Bar 1 and Bar 4 both having the DOFS bonded with silicone. Bar 3 and Bar 6, despite sharing the same DOFS-bonding adhesives, they share a total number of SRAs which are similar to respectively Bar 5 (1304 vs. 1339 SRAs) and to Bar 4 (858 vs. 737 SRAs). Such is a demonstration that the performance of combined adhesives has a substantial level of sporadicity that oscillates between the behavior of their constituents, in this case between cyanoacrylate and silicone. These are the only conclusions possible on (DM-AD1).

Analyzing now the SRAs amount per bar section it is evident that Bar 1 and Bar 4 (Silicone) have lower peaks than the rest suggesting that, per single DOFS coordinate, the number of anomalies are inferior compared to the others therefore guaranteeing more strain readability. When comparing Bar 4, 5, 6, the ones that include silicone hardly present any SRAs in two of the three incision while Bar 5 clearly has a large number of them around the incisions and the mid-span. This was possible thanks the intrinsic ability of the silicone to redistribute stresses therefore preventing their concentration in particular DOFS coordinates (as suggested by DM-AD4) that could lead up to the SRAs. Therefore, from a resistance point of view (intended as absence of anomalies), the silicone is the best adhesive. So much so that, among all the DOFS-instrumented rebars, the ones using silicone present an inferior number of total SRAs both in singular DOFS-coordinated and on the whole bar segment. The stress redistribution mechanism, despite slightly altering the strain readings, also delays the rise of anomalies (as evident in Figure 15) to higher strain levels. In addition, the silicone does not run the risk of cracking as other non-elastic adhesives [24]. This represents an advantage of the silicone over cyanoacrylate, not a limitation of the latter as reliable measurements can be taken also with cyanoacrylate until high strain levels, removing DM-AD4 form the possible causes of the rise of SRAs.

Summing up the points extracted above, it can be stated that there is not a better adhesive for any DOFS-instrumented test in RC members but rather a better one according to the objectives of the test itself. If high levels of precision in strain calculation are required, then the best bonding adhesive is cyanoacrylate, even though its fragility makes it more predisposed to having SRAs earlier on in experimental tests. On the other hand, if the main concern of the investigator lies in extending the anomaly-free monitoring duration time, then the optimal adhesive is silicone. While the strain measurements will be less precise, the number of anomalies will be inferior and the SRAs will start appearing later compared to other adhesives. Finally, if the main concern of the researcher is the protection of the DOFS from concrete friction, then the use of two layers of adhesives (cyanoacrylate and silicone) provides such with a hybrid behavior of its two base components.

Concerning disruption mechanisms DM-AD2 and DM-AD3 no apparent debonding was evident while only a small crack in cyanoacrylate was spotted. Yet, no anomalistic correlation can be found corresponding to the cracked section suggesting that only the most superficial layer of the adhesive had cracked, leaving intact the one below in direct contact with the DOFS. As a consequence of such, assuming a correct positioning of the adhesives these two disruption mechanisms can be discarded from the possible causes for the rise of SRAs.

## 4. Conclusions

The experimental test, topic of the present paper, demonstrated once again the large potential of DOFS for strain measurements on/inside civil engineering structures. The strains measured were indeed congruent both with the expected theoretical response and with the strains measured in parallel by the strain gauges, at stress levels of small and large entity (more than double of the yielding stress) and in a completely distributed manner. The Strain-Reading Anomalies, above defined as SRAs, are DOFS-produced strain readings glitches that prevent the extrapolation of useful data. The paper has given an interpretation of their possible causes focusing strictly on the physical aspect of the substrate on which the fiber is bonded. The results of such research, combined with studies on SRAs from a computational and optical point of view, can provide the groundings for a global and complete understanding of the SRA phenomenon. To study such physical-induced anomalies, an experimental campaign was designed to trigger them in an isolated environment to identify their real origin. The test consisted of gradually bending seven rebars (presenting different adhesives, varying transversal section, etc.) monitored by an ODiSI-A OBR machine manufactured by LUNA. The test results therefore have absolute validity only for the present OBR despite providing results pertinent to an issue occurring similarly for other commercial OBRs. Of the seven possible hypothesized physical causes for the rise of SRAs, named Disruption Mechanisms (DM), the only ones that were not excluded by the present test are the following:

- (DM-SB1) The surpassing of a Strain Anomaly Threshold (AST) beyond which the constitutive material of the fiber cannot bear any more stresses without showing anomalies in the readings. On the other hand, it also possible that, when the fibers are already under stress, they may display a high sensibility to variations in the mechanical behavior of its support that could lead up to SRAs.
- (DM-SB2) The present test was designed in such a way that only minor impact loads could be applied. Therefore, to correctly simulate the peaking of stresses that rebars experience whenever a crack opens in a RC member, further experimentation is required with higher impact loads or actual cracking concrete.
- (DM-AD1) The absence of concrete in the test prevented from checking the role that the friction of cracking concrete against the DOFS has in the rise of SRAs.

An experimental campaign will follow up the present one by studying the influence of the remaining disruption mechanisms on RC tie members. The paper’s experiment helped shedding light on the anomalistic behavior of DOFS. The following conclusions were extrapolated:

- The SRAs tend to rise in multiple neighboring DOFS coordinates forming Anomalistic areas.
- The anomalistic areas focus around sections of the rebar that are heavily stressed or that present a high strain gradient (such as the mid-span and the incised sections).
- The SRAs can be distinguished between Harmless SRAs (HL-SRAs) and Harmful SRAs (HM-SRAs) of which only the latter represents a serious threat for the investigators. Indeed, in the case of the first type of anomalies, following some anomalistic measurements are still multiple reliable ones making the HL-SRAs more of a nuisance than limit.
- Even though some HL-SRAs seem to appear earlier, most SRAs and all the HF-SRAs occur beyond a specific moment in the test which sees simultaneously the reaching of the strain value of roughly 4000 με (possible AST) in all the bars of the specimen and the yielding of the fixed ends of the bars causing a variation in the specimen’s load-deformation behavior. Only one or both these elements seem to have triggered the rise of SRAs.
- Different adhesives for the bonding of the fiber to the structural element were studied to evaluate their performance during the test. In particular, the adhesives used were cyanoacrylate, silicone and a combination of the two. The outcome of the test sees silicone to be the most resistant adhesive therefore ensuring a delayed appearance if SRAs and in smaller number to the expense of the precision of the strain measurements. Oppositely, the rigid but fragile nature of the cyanoacrylate provides the results with highest precision to the expense of resistance and endurance. In fact, the SRAs seem to spring up earlier and in bigger quantity in the latter. Meanwhile the combination of the two gives birth to a hybrid behavior. Regarding the adhesives, it was concluded that there was not a particularly better one, but it was rather a function of the objectives of the researcher.

## Figures and Tables

**Table 1:** DOFS coordinate of the first and last point bonded to each steel rebar and of load application.

Bars	First DOFS Coordinate Bonded to the Bar (m)	DOFS Coordinate of the Load Application Point of Each Bar (m)	Last DOFS Coordinate Bonded to the Bar (m)
1	1.36	1.57	1.85
2	1.99	2.28	2.56
3	2.67	2.96	3.24
4	3.37	3.63	3.88
5	4.00	4.28	4.57
6	4.65	4.94	5.21

**Table 2:** Percentage of SRAs over the total number of strain measurements below specific strain values.

**Strain Level (με)**	**Total Number of Strain Readings**	**Total Number of SRA-Free Strain Readings**	**Percentage of Readings with SRAs**	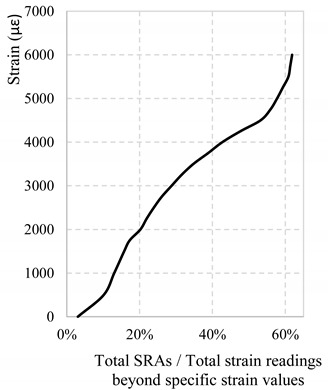
500	169,070	151,913	10.15%	
1000	131,553	114,411	13.03%	
1500	108,452	91,361	15.76%	
1750	98,605	81,560	17.29%	
2000	89,367	72,389	20.22%	
2250	81,819	64,878	21.95%	
2500	74,560	57,675	23.90%	
2750	67,725	50,906	26.08%	
3000	60,820	44,047	28.78%	
3250	55,015	38,262	31.52%	
3500	49,346	32,631	34.75%	
3750	43,330	26,748	38.82%	
4000	38,731	22,259	42.71%	
4250	33,891	17,609	47.68%	
4500	29,560	13,496	53.23%	
4750	27,523	11,698	55.93%	
5000	26,156	10,557	57.73%	
5250	25,210	9697	59.31%	
5500	24,303	8899	60.85%	
5750	23,965	8622	61.37%	
6000	23,639	8375	61.87%	
